# The Role of Genetically Engineered Probiotics for Treatment of Inflammatory Bowel Disease: A Systematic Review

**DOI:** 10.3390/nu15071566

**Published:** 2023-03-24

**Authors:** Tao Zhang, Jindong Zhang, Liping Duan

**Affiliations:** Department of Gastroenterology, Peking University Third Hospital, Beijing 100191, China

**Keywords:** inflammatory bowel disease, colitis, genetically modified probiotics, efficacy

## Abstract

Background: Many preclinical studies have demonstrated the effectiveness of genetically modified probiotics (gm probiotics) in animal models of inflammatory bowel disease (IBD). Objective: This systematic review was performed to investigate the role of gm probiotics in treating IBD and to clarify the involved mechanisms. Methods: PubMed, Web of Science, Cochrane Library, and Medline were searched from their inception to 18 September 2022 to identify preclinical and clinical studies exploring the efficacy of gm probiotics in IBD animal models or IBD patients. Two independent researchers extracted data from the included studies, and the data were pooled by the type of study; that is, preclinical or clinical. Results: Forty-five preclinical studies were included. In these studies, sodium dextran sulfate and trinitrobenzene sulfonic acid were used to induce colitis. Eleven probiotic species have been genetically modified to produce therapeutic substances, including IL-10, antimicrobial peptides, antioxidant enzymes, and short-chain fatty acids, with potential therapeutic properties against colitis. The results showed generally positive effects of gm probiotics in reducing disease activity and ameliorating intestinal damage in IBD models; however, the efficacy of gm probiotics compared to that of wild-type probiotics in many studies was unclear. The main mechanisms identified include modulation of the diversity and composition of the gut microbiota, production of regulatory metabolites by beneficial bacteria, reduction of the pro- to anti-inflammatory cytokine ratio in colonic tissue and plasma, modulation of oxidative stress activity in the colon, and improvement of intestinal barrier integrity. Moreover, only one clinical trial with 10 patients with Crohn’s disease was included, which showed that *L. lactis* producing IL-10 was safe, and a decrease in disease activity was observed in these patients. Conclusions: Gm probiotics have a certain efficacy in colitis models through several mechanisms. However, given the scarcity of clinical trials, it is important for researchers to pay more attention to gm probiotics that are more effective and safer than wild-type probiotics to facilitate further clinical translation.

## 1. Introduction

Inflammatory bowel disease (IBD), largely classified as Crohn’s disease (CD) or ulcerative colitis (UC), is a chronic intestinal inflammatory disorder mediated by genetic, immune, microbial, and environmental factors [1]. However, its precise etiology has not yet been clarified [2]. Over the last few decades, the incidence of IBD has risen rapidly not only in Western countries [3] but also in Asian countries such as China and India, entailing a growing socioeconomic burden [4]. Considering that chronic inflammation in the gastrointestinal tract (GIT) is ultimately a dysregulated and overactive immune response to the destruction of the intestinal environment in the host, immune abnormalities, such as adaptive and innate immunity, have been mostly explored in the investigation of IBD pathogenesis [2]. Consequently, in addition to conventional therapies such as 5-ASAs, antibiotics, and steroids, immunosuppressive drugs, including immunomodulators and biologics, are widely used in an attempt to regulate compromised immune homeostasis and achieve positive efficacy in the treatment of many IBD patients [5]. However, the effectiveness of these drugs is individually specific and may be limited by their short half-life in vivo, instability in the upper GIT, and systemic side effects caused by intravenous and subcutaneous administration.

Probiotics are live microorganisms similar to beneficial bacteria that are naturally present in the human GIT and provide beneficial health effects when orally administered in adequate amounts [6]. To date, they have been widely used to prevent and treat various medical conditions, including IBD, irritable bowel syndrome, *Helicobacter pylori* infections, *Clostridium difficile* infections, antibiotic-associated diarrhea, Parkinson’s disease, and even cancer [7,8,9]. The probiotic cell-free supernatant has also attracted attention as a safe and targeted alternative therapy and has been reported to alleviate indomethacin-induced colitis resembling Crohn’s disease by downregulating the inflammatory response and oxidative stress [10]. In recent years, probiotics have been shown to be effective vehicles for the delivery of therapeutic substances to treat specific conditions [11], which has raised the opportunity for researchers to genetically modify them to develop more pragmatic probiotics that produce and deliver IBD therapeutic proteins to the GIT locally, with lower cost, greater effectiveness, and fewer side effects than conventional immunosuppressive drugs administered by injection [12].

Although many in vivo and in vitro experimental studies have suggested the possible effectiveness of gm probiotics in different animal models of IBD, there is still little evidence of their effects compared with those of wild-type probiotics. Hence, we conducted this systematic review to summarize the efficacy of different gm probiotics compared to wild-type probiotics in the treatment of IBD in animal models and patients and to investigate the specific effects and main mechanisms involved. Additionally, a critical assessment of preclinical and clinical experiments was conducted to identify their methodological weakness to serve as guidance for future research.

## 2. Methods

### 2.1. Literature Search

The PubMed, Web of Science, Cochrane Library, and Medline databases were searched systematically from their inception to 18 September 2022, by two independent researchers to identify related preclinical and clinical studies. The lists of references from the included studies were manually searched to identify other possible studies. The present study was designed and conducted in line with the recommendations included in the Preferred Reporting Items for Systematic Reviews and Meta-Analyses (PRISMA) statement [13]. This systematic review was registered in PROSPERO, and the registration ID is CRD42022351738.

### 2.2. Eligibility Criteria

In this systematic review, the PICOS strategy was used to identify studies that met the following inclusion criteria:

1. Population: rodents with colitis and patients with IBD;
2. Intervention: supplementation with gm probiotics;
3. Comparisons: placebo; wild-type probiotics, etc.;
4. Outcomes: weight loss, colon length, disease activity, intestinal damage, anti- and pro-inflammatory cytokines, oxidative stress-related indicators, mucosal barrier function, etc.
5. Study design: preclinical studies, randomized controlled trials, cohort studies, etc.

### 2.3. Exclusion Criteria

The exclusion criteria included the following:

1. Duplicated studies;
2. In vitro studies or studies not related to our research topic;
3. Papers published in a language other than English;
4. Publication type: reviews, meta-analyses, and consensus papers
5. Papers without the data we focused on or without full text.

### 2.4. Data Extraction and Risk of Bias

Two authors independently extracted the following variables from each preclinical study: first author, publication year, country, experimental model features (lineage, sex, age, and type of colitis), research method (number of groups, number of animals per group, wild-type probiotics, constructed plasmid, recombinant probiotics, therapeutic substances, administration method, dose, and duration), and the outcomes mentioned in Section 2.2. For clinical studies, variables including the first author, publication year, country, population features (sex, age, and number of participants), experimental design, intervention (gm probiotics, dose and frequency of administration, and duration), and main outcomes were collected.

The criteria set in Animal Research: Reporting In Vivo experiments (ARRIVE) guidelines [14] were used to evaluate the risk of bias in preclinical studies in vivo, and the criteria proposed by Downs and Black were used to assess the risk of bias in clinical studies [15]. The quality of the clinical studies was classified by total scores as poor (≤4 of 13 points), intermediate (5–8 of 13 points), or good (≥9 of 13 points).

If there were any inconsistencies in the process of data extraction and the risk of bias assessment, the two authors discussed these issues, or an independent expert in this field was consulted to reach a consensus.

## 3. Results

### 3.1. Study Selection

**Figure 1:**
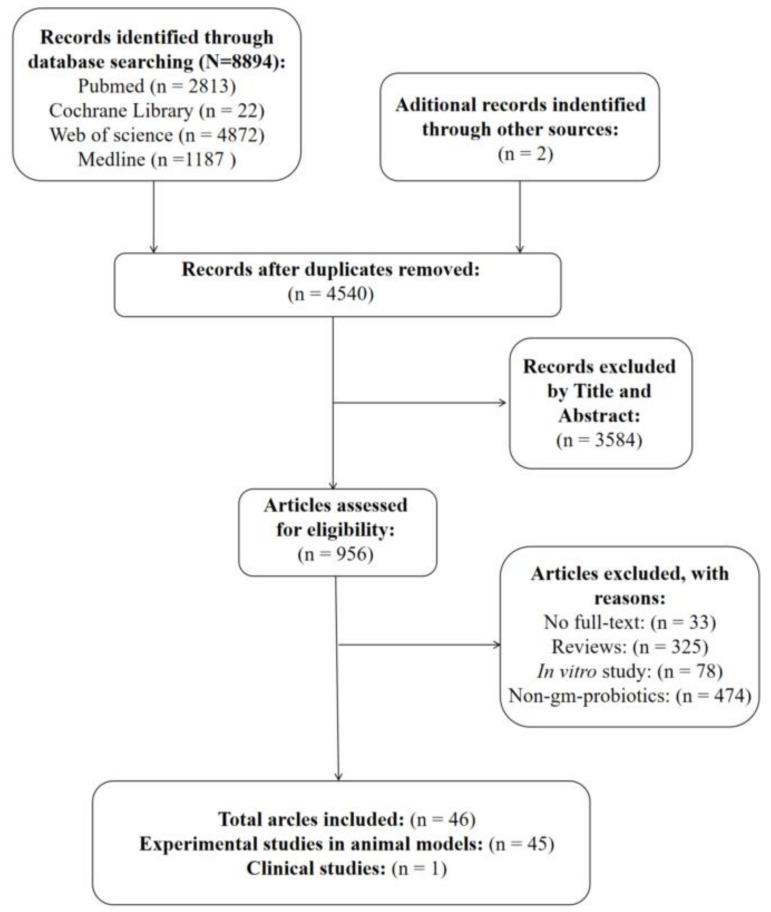
Flow diagram of the literature search and screening process.

As shown in Figure 1, 8894 records were identified in the initial database search. A manual search of the reference lists yielded two additional studies. After removing duplicates, 4540 studies were reviewed by titles and abstracts, of which 3584 studies were excluded because they were not associated with our research topic. The full texts of the remaining 956 papers were further screened, and 910 articles were excluded; the reasons for this are shown in Figure 1. Finally, 45 preclinical studies [16,17,18,19,20,21,22,23,24,25,26,27,28,29,30,31,32,33,34,35,36,37,38,39,40,41,42,43,44,45,46,47,48,49,50,51,52,53,54,55,56,57,58,59,60] and 1 clinical study [61] fulfilled the inclusion criteria for this systematic review.

### 3.2. Qualitative Data

The general characteristics of all the included preclinical studies are presented in Table 1. All eligible preclinical studies were published after 2000 and were conducted in 13 countries. The animal lineages used for modeling included BALB/c mice (*n* = 19), C57BL/6 mice (*n* = 22), Wistar rats (*n* = 2), and Sprague Dawley (SD) rats (*n* = 2). Most studies used male animals (*n* = 23), and only two studies used both male and female animals; four studies did not report sex. The age of the animals ranged from 2 to 20 weeks, although four studies did not report this information. Regarding the colitis models, dextran sulphate sodium (DSS) (*n* = 34) was most commonly used to induce colitis, followed by trinitrobenzene sulfonic acid (TNBS) (*n* = 10), dinitro-benzenesulfonic acid (DNBs) (*n* = 2), anti-CD3 antibody (*n* = 1), IL-10 knockout (IL-10^−/−^) (*n* = 4), and T-cell transfer (*n* = 1); interestingly, five studies used more than one type of colitis model. 

In these experimental studies, 11 different probiotic species, including *Lactococcus lactis* (*n* = 20), *Escherichia coli* (*n* = 7), *Streptococcus thermophilus* (*n* = 2), *Lactobacillus casei* (*n* = 4), *Lactobacillus paracasei* (*n* = 1), *Bacteroides ovatus* (*n* = 1), *Saccharomyces boulardii* (*n* = 1), *Lactobacillus fermentum* (*n* = 2), *Bifidobacterium longum* (*n* = 5), *Lactobacillus plantarum* (*n* = 1), and *Saccharomyces cerevisiae* (*n* = 2) were used as chassis to be genetically modified to secrete different therapeutic substances with potential properties against colitis. In all studies except one, the animals received gm probiotics via gastric gavage at doses ranging from 10^5^ to 4 × 10^12^ colony-forming units (CFUs)/day, although one study did not report the dose. Finally, the duration of the intervention ranged from 3–6 weeks (Table 2).

Only one human study [61] performed in the Netherlands was included. This study was a placebo-uncontrolled trial in 2006, with 10 CD patients receiving 10 capsules of 1 × 10^10^ CFU of genetically modified *L. lactis* producing IL-10 (LL-Thy12) twice daily for 7 days.

We focused on the efficacy of gm probiotics relative to wild-type probiotics in treating patients with IBD or animal models, unless these data were not accessible, in which case only the properties against colitis were described. The extracted data are presented in Supplementary Material S1.

### 3.3. The Efficacy of Gm Probiotics Secreting Immunoregulatory Cytokines on Colitis Models and IBD Patients

#### 3.3.1. IL-10

Twelve preclinical studies [16,17,21,26,27,28,29,36,46,48,49,58] engineered probiotics to secrete IL-10. Of these studies, disease activity [48,49] and colon length [21,48,49] were assessed in only two and three studies, respectively, with a significant improvement in the gm probiotics group compared to the group receiving wild-type probiotics or the untreated group. Relative body weight presented inconsistent or even contradictory results, with either increases [26,29,58], decreases [21], or no changes [21,27,36] in the group using gm probiotics compared to the group using wild-type probiotics. As for intestinal damage, we were able to capture a relatively consistent trend that gm probiotics producing IL-10 can reduce intestinal damage observed macroscopically compared to wild-type probiotics in most studies.

Cytokine profile analysis generally showed that gm probiotics promoted higher IL-10 expression in the colon or serum than that of the wild-type probiotics. Although the levels of the other cytokines were reported without any consistent results, it is notable that *B. longum* producing IL-10 elicited higher suppression of the levels of IFN-γ, TNF-α, IL-1β, and IL-6 compared to the wild-type strain [48]. Three studies [27,28,46] reported the activity of colonic myeloperoxidase (MPO), a marker of neutrophil infiltration and oxidative stress, but only one study showed a significant improvement [28].

Intriguingly, a clinical study [61] with only ten CD patients showed that treatment with LL-Thy12 was safe, with minor adverse events, and a decrease in disease activity was observed in these patients, indicating that the use of gm probiotics for the mucosal delivery of IL-10 might be a feasible strategy for treating IBD. However, given the small sample size and limited outcome measures, these results should be interpreted with caution.

#### 3.3.2. IL-27

IL-27, a pleiotropic cytokine belonging to the IL-12 family, has immunosuppressive and therapeutic effects in colitis [62]. Hanson et al. [50] developed a strategy for delivering IL-27 to the GIT by genetically modifying *L. lactis* to synthesize bioactive IL-27 (LL-IL-27) in situ. In this study, compared with *L. lactis*, oral administration of LL-IL-27 showed a stronger protective effect against CD4^+^CD45RB^hi^ T-cell transfer-induced colitis by alleviating intestinal damage and promoting IL-10 expression in the colon.

#### 3.3.3. IL-35

Two studies [32,35] constructed recombinant *E. coli* and *L. lactis* strains expressing IL-35. Both have a greater ability to alleviate intestinal damage and improve the disease activity index (DAI) score and colon length than wild-type probiotics. Additionally, they have been shown to modulate the expression of anti- and pro-inflammatory cytokines in the colon and plasma of colitis models [35] and have a higher ability to suppress IL-6 and increase IL-10 levels than wild-type probiotics [32].

#### 3.3.4. Growth Factors

Growth factors play a key role in intestinal growth, regeneration, damage repair, and immunoregulation; however, it is difficult to achieve therapeutic functions with oral administration owing to their instability in the upper GIT. A study by Hamady et al. [23] explored the gm probiotics producing keratinocyte growth factor-2 (KGF-2) or transforming growth factor-beta (TGF-β), which showed a significant prophylactic effect on limiting the development of intestinal inflammation in comparison to wild-type probiotics. Additionally, oral administration of recombinant *L. lactis* expressing insulin-like growth factor I (IGF-I) improved intestinal damage and barrier integrity and reduced colonic MPO activity in DSS-induced colitis mice; however, when compared with wild-type probiotics, it only achieved better alleviation of the histological damage score [53].

#### 3.3.5. Other Immunoregulatory Cytokines

Trefoil factors (TFFs), a family of human cytokines known to promote intestinal barrier function and epithelial restitution, do not yield therapeutic outcomes in IBD after oral delivery, as they adhere strongly to the mucus layer of the small bowel [63]. To overcome this limitation, Praveschotinunt et al. [34] used *E. coli* Nissle 1917 (EcN) as a vehicle to produce curli fibrous matrices displaying and tethering TFF3, which showed enhanced protective effects against acute colitis for rectal administration; however, these effects were not significant when compared to the parental strains.

The efficacy of gm probiotics in colitis differed not only in terms of the therapeutic substances it produced, but also the phases of administration; for instance, a short and early administration of recombinant *L. lactis* producing thymic stromal lymphopoietin (TSLP), a cytokine in mature dendritic cells with properties of inhibiting IL-12 secretion and inducing differentiation of anti-inflammatory FoxP3+ Treg, was more effective than a long-lasting treatment [60].

#### 3.3.6. Antibodies or Receptor Antagonist for Pro-Inflammatory Cytokines

Interventions targeting pro-inflammatory cytokine signaling have also been demonstrated as an effective approach in several animal studies [46,64]. However, they have been reported to cause serious side effects when administered intravenously or subcutaneously in clinical patients; thus, local delivery of these therapeutic substances to the GIT is desirable to restrict these side effects [65].

A study by Chiabai et al. [33] using *L. lactis* as a vehicle for delivering anti-TNFα to GIT of acute colitis model showed a higher efficacy than *L. lactis*. Moreover, compared to *L. lactis* and *L. lactis* producing IL-10, it exerted a greater ability to alleviate chronic colitis induced by DSS and IL-10^−/−^ [46]. Similarly, Namai et al. [37] used *L. lactis* as a chassis for the delivery of IL-1Ra to the intestinal mucosa, which showed a significantly higher efficacy in suppressing disease activity in mice with acute colitis than with wild-type probiotics. These results suggest that a novel, effective, and inexpensive IBD therapy that blocks pro-inflammatory cytokine signaling has been successfully developed.

#### 3.3.7. Comparisons of Different Gm Probiotics

Interestingly, two studies [28,36] have compared the efficacy of different gm probiotics, with one study [28] using probiotics producing anti-inflammatory cytokines (IL-10 and TGF-β1) and serine protease inhibitors (Elafin and SLPI) and another using [36] probiotics secreting IL-10, TNFR1-ECD, alkaline phosphatase (AP), and atrial natriuretic peptide (ANP). Finally, the results demonstrated that *L. lactis* secreting serine protease inhibitors and *S. boulardii* secreting ANP may be the most effective probiotics for the treatment of colitis.

### 3.4. The Efficacy of Gm Probiotics Secreting Antioxidant Enzymes on Colitis Models

Eight studies [18,20,24,25,31,47,51,56] have successfully expressed catalase (CAT) or superoxide dismutase (SOD) in different probiotics, with prominent antioxidant activity.

*L. fermentum* P126 producing CAT showed superior effects in improving the intestinal damage, reducing the activity of lipid peroxidation and MPO, as well as activating NF-κB in colon tissue when compared to wild-type probiotics [24]. Consistently, the administration of SOD-secreting gm probiotics also revealed a significant improvement in intestinal damage, inflammation, and oxidative stress in colitis models [18,56], and compared to wild-type probiotics, *B. longum* [31] and *L. fermentum* [51] producing SOD exerted a higher efficacy.

Three publications [20,25,47] focused on both CAT- and SOD-producing probiotics. LeBlanc et al. [20] found that mice with acute colitis receiving CAT- or SOD-producing *L. casei* BL23 showed faster recovery from weight loss and increased SOD and CAT activities in the colon compared to mice receiving the wild-type strain. Interestingly, CAT- and SOD-producing *S. thermophilus* CRL 807 were administered to mice with chronic colitis as a suspension in saline solution or in fermented milk in a study conducted by del Carmen et al. [25], which revealed that these gm probiotics in fermented milk were more effective than in saline solution; for instance, the former significantly alleviated intestinal damage compared to the parental strain, but the latter showed almost no superiority. Furthermore, beneficial effects were improved in mice receiving a mixture of both CAT- and SOD-producing *S. thermophilus* CRL807; however, these effects were not obvious in a study performed by Watterlot et al. [47].

Heme oxygenase-1 (HO-1) is an antioxidant enzyme induced by inflammatory stimuli and oxidative stress [66]. Only one study [30] showed that oral administration of *L. lactis* NZ9000 secreting HO-1 significantly alleviated colitis-associated symptoms, histological damage, and immune disorders in mice compared to *L. lactis* NZ9000 with an empty vector.

### 3.5. The Efficacy of Gm probiotics Secreting Antimicrobial Peptide on Colitis Models

Antimicrobial peptides such as defensins and cathelin-related antimicrobial peptide (CRAMP) are protective factors that constitute complex chemical barriers on the layer of continuous epithelial cells in the GIT [67]. Increased expression of antimicrobial peptides in colonic mucosa has been reported in response to inflammation and infection [22], indicating an essential role in immune regulation and wound healing. However, their short half-lives and sensitivity to acidic environments in the GIT greatly limit their clinical application in IBD therapy [40].

Four studies engineered *L. lactis* NZ3900 [22,38,40,57] to produce antimicrobial peptides. Of these studies, two [22,40] focused on CRAMP-producing *L. lactis*, confirming its effectiveness in preventing and attenuating colitis, especially at 10^10^ CFU daily [22]. Likewise, the defensin-5-secreting *L. lactis* can also alleviate mucosal damage by suppressing NF-κB signaling pathway [38].

Pancreatitis-associated protein (PAP), a C-type lectin belonging to the regenerating islet-derived III protein family, plays a protective role in colitis. Breyner et al. [57] tested the efficacy of *L. lactis* secreting PAP (LL-PAP) in DNBS- and DSS-induced colitis, and protective effects were detected only in the DNBS colitis model. Moreover, compared to *L. lactis*, LL-PAP significantly improved the gut microbial composition, especially that of butyrate-producing bacteria such as *Eubacterium plexicaudatum.*

### 3.6. The Efficacy of Gm Probiotics Promoting Production of Short-Chain Fatty Acids (SCFAs) or Related Organic Acids in GIT

SCFAs, produced by bacteria that ferment fibers in the GIT, are carboxylic acids with aliphatic tails of 1–6 carbons, of which acetate, propionate, and butyrate are the most abundant [68]. Due to their anti-inflammatory properties, gut barrier protection, and immunomodulation, SCFAs are unfortunately reported to be typically reduced in the feces and gut mucosa of patients with IBD [68].

Park et al. [41] engineered *E. coli* MG1655 (MG1655-BCD-BUT) and *EcN* (EcN-BCD-BUT) to produce butyric acid and showed a significant amelioration of the DAI score and lower expression of IL-6 and MPO in the colonic tissue of DSS-induced colitis models. In particular, compared with MG1655-BCD-BUT, EcN-BCD-BUT alleviated intestinal damage and inflammation more significantly, proving its potential superiority.

These gm probiotics have shown great effects in improving gut microbiota homeostasis. For instance, *S. cerevisiae* secreting lactic acid appeared to improve α-diversity and decrease the *Firmicutes* to *Bacteroides* ratio in DSS-induced colitis mice [43]. Yan et al. [45] demonstrated that compared with *EcN*, oral application of *EcN* producing (R)-3-hydroxybutyrate (EcNL4) was more effective at improving mouse weight, colon length, histological damage, MPO activity, and SCFA levels in the colon, as well as serum cytokine levels. Furthermore, the abundance of *Akkermansia* and *Prevotella* significantly increased in the EcNL4 group.

Notably, the genetically engineered *EcN* highly expressed *schistosome* immunoregulatory protein Sj16, which was found to promote the growth of *Ruminococcaceae* in the GIT and therefore enhance the production of butyrate, mediating the attenuation of the disease activity of colitis [44]. This is an excellent example of a gm probiotic indirectly promoting SCFA production.

### 3.7. The Efficacy of Gm Probiotics Secreting Alpha-Melanocyte-Stimulating Hormone (α-MSH) on Colitis Models

α-MSH is a neuropeptide that elicits anti-inflammatory properties in various disease models, including IBD and arthritis. However, its clinical application is limited because of its extremely short duration in vivo; thus, probiotics might be effective carriers to facilitate efficient oral delivery of α-MSH to address this limitation [69].

α-MSH-secreting *L. casei* showed a significant effect on attenuating acute colitis as assessed by body weight loss, intestinal damage score, MPO activity, pro- and anti-inflammatory cytokines levels, and survival rate; however, its efficacy relative to wild-type probiotics is unknown [19]. Subsequently, two studies [54,55] utilizing α-MSH-producing *B. longum* against DSS-induced acute colitis revealed that *B. longum*-α-MSH was more effective than *B. longum*.

### 3.8. The Efficacy of Gm Probiotics Secreting Other Therapeutic Substances on Colitis Models

Palmitoylethanolamide (PEA), produced by the conjugation of palmitate and ethanolamine through N-acyl-phosphatidylethanolamine-specific phospholipase D (NAPE-PLD) [70], harbors properties of modulating several physiological processes, including analgesia, neuroprotection, and inflammation [71]. By activating the peroxisome proliferator–activated receptor-α (PPARα), PEA exerts potent anti-inflammatory effects to improve intestinal inflammation [72]. However, even though it is safe without serious side effects, high doses are required to achieve therapeutic effects, which limits its use in current clinical practice. To overcome this limitation, a study [39] has genetically modified *L. paracasei* F19 to secrete NAPE-PLD (pNAPE-LP), which can release PEA from the GIT in the presence of ultra-low doses of exogenous palmitate. The results revealed significant amelioration of colitis in the group administered with pNAPE-LP.

Extracellular adenosine triphosphate (eATP), produced by commensal microbiota and immune cells in the host, activates purinergic signaling via purinergic receptors, boosting inflammation and pathological damage in the intestine. Thus, purinergic signaling is a potential therapeutic target in IBD [42]. Scott et al. [42] developed a self-tunable *S. cerevisiae* (APTM-3) which secreted the CD39-like eATP-degrading enzyme apyrase when engineered human P2Y2 receptors detected eATP. The researchers used three colitis models: TNBS-, DSS-, and anti-CD3 antibody-induced colitis, which showed significantly suppressed intestinal inflammation and damage, reduced fibrosis, and improved gut microbiota in the APTM-3 group compared to the wild-type strain group.

Secretions from pathogens such as parasitic nematodes and pathogenic yersiniae can modulate host immune responses to induce an anti-inflammatory environment that favors their persistence and reproduction in hosts. Thus, these molecules can be viewed as potential therapeutic agents for IBD. Whelan et al. [52] demonstrated stronger anti-inflammatory properties of recombinant *EcN* secreting cystatin from the rodent nematode *Acanthocheilonema viteae* (AvCys) than those from *EcN*. Consistently, oral administration of recombinant *L. lactis* producing a low-calcium response V (LcrV) protein from the enteropathogenic species *Yersinia pseudotuberculosis* also showed prominent efficacy in both TNBS- and DSS-induced models, in contrast with *L. lactis* [59]. Additionally, TNBS-induced colitis was not prevented in IL-10^−/−^ mice, indicating that IL-10 is required for LcrV-mediated protection against IBD.

### 3.9. Risk of Bias

All the included studies had sufficient scientific contextualization and objectives. Eight studies did not provide ethical statements. Twenty-six studies provided information regarding whether the experiments were performed in a blind-controlled manner. None of the studies reported the time of day chosen for treatment administration, the rationale for choice of a specific route of administration, an explanation regarding the decision of animal numbers and details of sample size calculation, or the order in which the animals in the different experimental groups were treated and assessed. Only one study has described the rationale for choosing a specific dosage; that is, 2 × 10^9^ CFU, which represents the technically maximum reliable dose for freshly cultured strains. Sixteen studies reported the animals’ weight ranges before the intervention. Thirty articles did not describe how animals were allocated to the experimental groups. Only 12 studies reported mortality rates.

Based on the criteria proposed by Downs and Black, the clinical study included in this systematic review was classified as intermediate quality. Therefore, further high-quality studies are required (Supplementary Material S1).

## 4. Discussion

**Figure 2:**
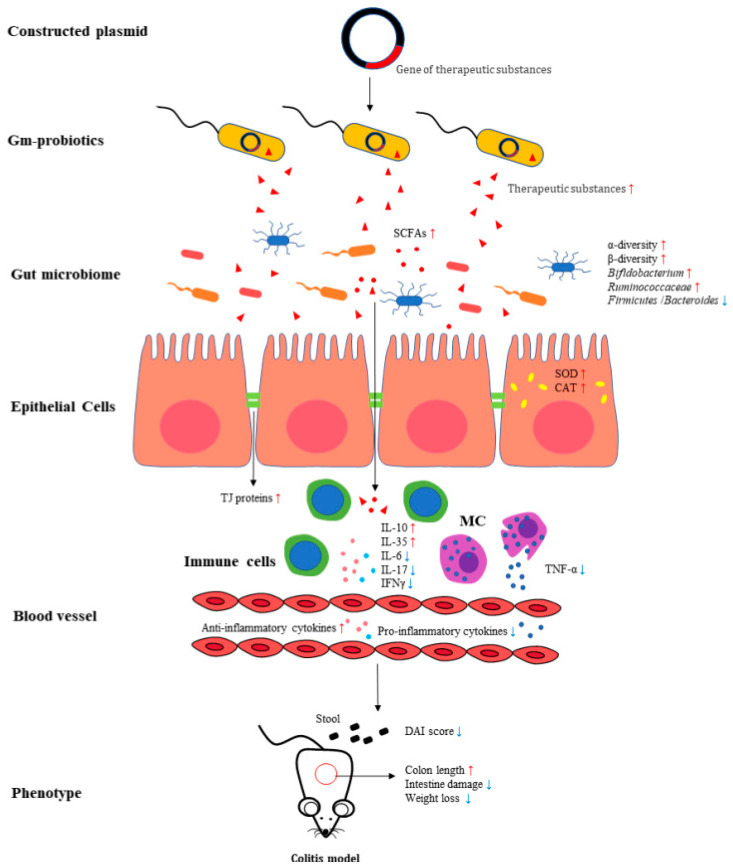
**The mechanism of gm probiotics involved in treating colitis models.** Constructed plasmids containing genes for therapeutic substances are integrated into wild-type probiotics to treat colitis animal models by gavage or rectal administration. The gm probiotics ameliorated the clinical phenotypes of colitis, such as DAI score, body weight loss, and intestinal damages, via the mechanisms involved in improving gut microbiota, increasing the level of short-chain fatty acids, regulating immune cells, reducing expression of the pro-inflammatory cytokines, increasing anti-inflammatory cytokines levels, and increasing the expression of tight junction proteins. (SCFAs: short-chain fatty acids; MC: mast cells; TJ proteins: tight junction proteins; DAI: disease activity index).

To the best of our knowledge, this is the first systematic review to summarize the efficacy of different gm probiotics in the treatment of IBD in animal models and patients, providing reference values for conducting and improving subsequent clinical research, and thus further improving the quality of life of patients with IBD. Despite the heterogeneity of these studies, our findings showed a certain effect of gm probiotics against colitis. Several protective mechanisms have been identified: reduction of the pro- to anti-inflammatory cytokine ratio in colonic tissue and plasma, modulation of the activity of oxidative stress in the colon, improvement of intestinal barrier integrity, modulation of the diversity and composition of gut microbiota, and production of favorable metabolites, including SCFAs, by beneficial bacteria. These mechanisms may contribute to the alleviation of phenotypes such as weight loss, colon length, disease activity, and intestinal damage in colitis models (Figure 2). Furthermore, it is notable that many studies have not reported the outcomes of the efficacy of gm probiotics compared to that of wild-type probiotics, making it difficult to further evaluate the properties of these gm probiotics. Only gm probiotics that are superior to wild-type probiotics can be regarded as successful.

Most studies were preclinical experiments, and only one phase I clinical trial [61] performed by Braat et al. in 2006, was included in this systematic review, which indicated that the barriers to using gm probiotics to treat IBD still exist in human studies. Several factors may contribute to the limited number of clinical studies: IBD patients are susceptible to intestinal microbiota translocation, leading to systemic sepsis. Thus, although the probiotics are generally regarded as safe, there still may be risks in the IBD population [73]. The colonization of the gm probiotics in the intestine was not sufficient to maintain the long-term efficacy; hence, the probiotics must be taken frequently, which may in turn increase the risk of side effects. The preclinical studies of some gm probiotics were scarce, and the laboratory data obtained were insufficient to support their clinical use and the limitations of IBD models. Thus, more well-designed preclinical studies and large-scale multicenter randomized controlled trials are needed to evaluate the beneficial effects and safety of gm probiotics in IBD.

There are many candidate therapeutic substances that have shown potential efficacy against colitis in previous studies; however, their further development to clinical applications have been limited by several shortages. For instance, as summarized in the Section 3, the growth factors were not stable in upper GIT, TFFs were reported to adhere strongly to the mucus layer of the small bowel, antibodies or receptor antagonist for pro-inflammatory cytokines were reported to cause serious side effects through intravenous or subcutaneous administration in clinical patients, and CRAMP and α-MSH showed a short half-life in vivo. Orally administered SCFAs have low bioavailability because they are efficiently absorbed by the upper GIT, reducing their therapeutic function in the lower GIT or colon of patients [74]. Additionally, SCFAs have a certain sour odor and poor palatability, which makes it particularly important to optimize the delivery methods of SCFAs, such as coating technology. The effectiveness of these administration methods for patients still needs to be confirmed by further research. Thus, using probiotics as a chassis to deliver therapeutic substances into the GIT can not only improve drug utilization, but also allows it to fully exert its therapeutic effect in local lesions while reducing systemic adverse reactions, which is beneficial for clinical application.

A wide variety of probiotics were used as chassis to deliver therapeutic substances into GIT, and given the properties of immunomodulation and surviving passage through the GIT, the species *L. lactis,* including *L. lactis* MG1363 and *L. lactis* NZ9000, was used most often [75]. The first evidence of this application in a colitis mouse model was found in an experiment performed by Steidler et al. in 2000 [16]; thereafter in 2006, *L. lactis* secreting IL-10 was used in clinical trials to treat Crohn’s disease and showed potential efficacy [61]. In addition to *L. lactis* and *E. coli*, the development of a new chassis in more dominant microorganisms from the gut microbiome is essential because these species may be more effective depending on their multiple properties, such as better adaptation to the human intestine, better colonization to achieve high cell numbers in the GIT, and safe interaction with the immune system [7]. However, there is still no consensus in the literature as to which is the most effective and active species or strain to be used as a chassis for delivering therapeutic molecules; thus, experiments comparing the biological activities of these probiotics are also needed. Moreover, the evaluation of the properties of gm probiotics or the colonization of wild-type strains in the intestine is important and has been ignored in many studies.

The dose of gm probiotics is still undefined and varies across studies, ranging from 10^5^ to 4 × 10^12^ CFU per day per mouse or rat in this systematic review, making it impracticable to suggest a specific dose. Few studies have simultaneously compared the efficacy of different concentrations of gm probiotics in the treatment of colitis. Qiu et al. [49] tested the effectiveness of three doses (2 × 10^7^, 2 × 10^8^, and 2 × 10^9^ CFU/mL) of recombinant *L. casei* CECT 5276 secreting IL-10, with the highest concentration (2 × 10^9^ CFU/mL) being the most effective. However, the dose-dependent effect of gm probiotics requires further validation in more studies. The time effect of gm probiotics on colitis models is undefined and worthy of exploration.

The survival rate of gm probiotics in GIT could improve when multiple complementary species are administered simultaneously. This may be because different parts of a genetic circuit in plasmids could be distributed among different species, increasing their co-dependence and relieving the metabolic strain [7]. Additionally, mixing several grams of probiotics that secrete different proteins may produce additional effects. In the study performed by del Carmen et al. [25], the greatest anti-inflammatory activity was observed in the group that received a mixture of both CAT- and SOD-producing *Streptococci*. In contrast, Watterlot et al. [47] observed that the combination of *Lb. casei* MnKat and *Lb. casei* BL23MnSOD showed no such effects. Despite the conflicting results of these two studies, this method still deserves to be used as a reference for subsequent studies as well as a promising treatment for IBD.

Many IBD models are available for use, and according to a review by Mizoguchi et al. [76], they can be classified into five major groups: the chemically induced model, the cell-transfer model, the spontaneous model, the congenital model, and the genetically engineered model. Chemically induced models such as DSS-induced- and TNBS-induced colitis were most frequently used to evaluate the efficacy of gm probiotics. Each model has specific advantages over others; for example, the DSS model was mainly used for exploring epithelial homeostasis/regeneration and wound healing processes, and IL10^−/−^ mice, which were also used in some studies, contributed to understanding the mechanisms of probiotics, *Helicobacter*, and NSAIDs in IBD. Thus, it is a general trend to utilize more than one colitis model to verify the function of gm probiotics in one study.

The risk of bias assessments in our systematic review demonstrated that much information related to study design and results was neglected or not reported by many studies; thus, improvements in the methodology and reporting of animal experiments are needed to ensure the quality and persuasiveness of studies in the future.

## 5. Conclusions

These findings indicate that several factors may affect the efficacy of gm probiotics, such as the species or strains of wild-type probiotics, different gm probiotic combinations, therapeutic substances, the dose, and the IBD model. Overall, gm probiotics have a certain effect on colitis models, which might be attributed to the following mechanisms: reduction of the pro- to anti-inflammatory cytokine ratio in the colonic tissue and plasma, modulation of the activity of oxidative stress in the colon, improvement of intestinal barrier integrity, modulation of the diversity and composition of the gut microbiota, and production of regulatory metabolites by beneficial bacteria. It is also important for researchers to pay more attention to gm probiotics, which are more effective and safer than wild-type probiotics, to facilitate clinical translation. Additionally, the methodology and reporting of animal experiments and clinical trials should be improved to ensure the quality of studies.

## Figures and Tables

**Table 1:** Characteristics of preclinical studies.

Author	Year	Country	Lineage	Sex *	Age (Week)	Number of Groups	Number of Animals	Model	Acute/Chronic Course
Del Carmen et al. [29]	2015	Argentina	BALB/C mice	Female	5	4	32	TNBS-induced colitis	Acute
Gardlik et al. [21]	2012	Slovak Republic	C57BL/6 mice	Male	10	6	60	DSS-induced colitis	Acute
Foligné et al. [17]	2006	France	BALB/C mice	Female	7–8	4	32–48	TNBS-induced colitis	Acute
Del Carmen et al. [26]	2014	Argentina	BALB/C mice	Female	5	5	90	TNBS induced colitis	Chronic
Martín et al. [27]	2014	France	C57BL/6 mice	Male	6–8	4	64	DNBs induced colitis	Chronic
Steidler et al. [16]	2000	Belgium	BALB/C mice	Female	N.A.	13	130	DSS induced colitis	Chronic
			129 SvIEv IL-10^−/−^ mice	Female	3–7	3	15	IL-10^−/−^ mice	Chronic
Hamady et al. [23]	2013	Britain	C57BL/6 mice	Male	8	7	56	DSS induced colitis	Acute
Bermúdez-Humarán et al. [28]	2015	France	C57BL/6 mice	N.A.	6–8	10	60–80	DSS induced colitis	Acute
Liu et al. [36]	2020	Taiwan	C57BL/6JNarl mice	Male	7–8	7	38	DSS induced colitis	Acute
Chiabai et al. [33]	2019	Brazil	C57BL/6 mice	Female	10	4	16–20	DSS induced colitis	Acute
Namai et al. [37]	2020	Japan	C57BL/6 mice	Female	7	2	36	DSS induced colitis	Acute
Zhang et al. [32]	2018	China	BALB/C mice	Male	6–8	4	40	DSS induced colitis	Acute
Wang et al. [35]	2019	China	C57BL/6 mice	Female	6–8	6	30	DSS induced colitis	Acute
Zhang et al. [24]	2013	China	BALB/C mice	Female	7	5	40	DSS induced colitis	Acute
Xie et al. [31]	2017	China	Wistar rats	Male	9–10	4	48	TNBS induced colitis	Acute
LeBlanc et al. [20]	2011	Argentina	BALB/C mice	Female	5	5	90	TNBS induced colitis	Acute
Del Carmen et al. [25]	2014	Argentina	BALB/C mice	Female	5	6	36	TNBS induced colitis	Chronic
Han et al. [18]	2006	France	Wistar rats	Male	N.A.	15	110	TNBS induced colitis	Acute
Wong et al. [22]	2012	China	BALB/C mice	Male	6–8	10	94	DSS induced colitis	Acute
Li et al. [40]	2021	China	C57BL/6 mice	Male	7–8	6	30	DSS induced colitis	Acute
Zeng et al. [38]	2020	China	C57BL/6 mice	Male	6–8	4	28	DSS induced colitis	Acute
Esposito et al. [39]	2021	Italy	C57BL/6J mice	Male	6	7	70	DSS induced colitis	Acute
Park et al. [41]	2021	Korea	C57BL/6J mice	Male	8	6	60	DSS induced colitis	Acute
Yan et al. [45]	2021	China	C57BL/6J mice	Male	7	5	25	DSS induced colitis	Acute
Yoon et al. [19]	2008	Korea	BALB/C mice	Female	6	4	20	DSS induced colitis	Acute
Shigemori et al. [30]	2015	Japan	C57BL/6 mice	Female	7	4	39	DSS induced colitis	Acute
Praveschotinunt et al. [34]	2019	USA	C57BL/6NCrl mice	Female	8–9	8	38–49	DSS induced colitis	Acute
Sun et al. [43]	2021	China	C57BL/6 mice	Male	6–8	4	26	DSS induced colitis	Acute
Scott at al [42]	2021	Canada	C57BL/6J mice	Male	8–10	4	36	TNBS induced colitis	Acute
				Female	8–10	4	41	DSS induced colitis	Chronic
				Female	8–10	5	29	Anti-CD3 antibody-induced enteritis	Acute
Wang et al. [44]	2021	China	BALB/C mice	Male	6	9	37–53	DSS-induced colitis	Acute
Wei et al. [54]	2016	China	SD rats	Male and Female	N.A.	4	48	DSS-induced colitis	Acute
Wei et al. [55]	2016	China	BALB/c mice	Male	6–12	4	40	DSS-induced colitis	Acute
Vandenbroucke et al. [46]	2010	Belgium	BALB/c mice	Female	11	5	50	DSS-induced colitis	Chronic
			IL10 knockout mice	N.A.	20	10	87	IL10 knockout mice	Chronic
Liu et al. [53]	2016	China	BALB/c mice	Female	8	5	40	DSS-induced colitis	Acute
Zurita-Turk et al. [58]	2020	Brazil	IL-10^−/−^ mice and wild-type mice	N.A.	2	4	About 36	IL-10^−/−^	Chronic
Qiu et al. [49]	2013	China	BALB/c mice	Female	4–6	8	64	DSS-induced colitis	Acute
Yao et al. [48]	2011	China	BALB/c mice	Male	6	5	50	DSS-induced colitis	Acute
Hanson et al. [50]	2014	USA	C57BL/6 and Rag1^−/−^	Male	7.5	N.A.	N.A.	Transfer of CD4 + CD45RBhi T cells-induced colitis	Chronic
Whelan et al. [52]	2014	Germany	C57BL/6 mice	Male	9–11	4	45	DSS induced colitis	Acute
Breyner et al. [57]	2019	France	C57BL/6 mice	N.A.	6–8	4	N.A.	DNBS-induced colitis	Acute
						4	N.A.	DSS-induced colitis	Acute
Liu et al. [56]	2018	China	SD rats	Male	N.A.	4	48	DSS-induced colitis	Acute
Hou et al. [51]	2014	China	BALB/c mice	Female	6	4	60	TNBS-induced colitis	Acute
Watterlot et al. [47]	2010	France	BALB/c mice	Male	7	5	50	DSS-induced colitis	Acute
Aubry et al. [60]	2015	France	C57BL/6 mice	N.A.	6	6		DSS-induced colitis	Acute
Foligne et al. [59]	2007	France	BALB/c and C57BL/6	Female	7–9	4	40	TNBS-induced colitis	Acute
						4	40	IL-10^−/−^ and TNBS-induced colitis	Acute
						8	80	DSS-induced colitis	Acute

* We only extracted information on the animals used to evaluate the efficacy and safety of gm probiotics.

**Table 2:** Methods used in preclinical studies.

Author	Year	Wild-Type Probiotic Strain	Constructed Plasmid with Function	Recombinant Probiotic Name	Secretions	Administration	Dose/Day	Length
del Carmen et al. [29]	2015	*S. thermophilus* CRL807	pValac::il-10	*S. thermophilus* CRL 807pValac::il-10	IL-10	Gastric gavage	10^8^ CFU	12 days
Gardlik et al. [21]	2012	*E. coli* Nissle 1917	pMEC-IL10	Nissle 1917/pMEC-IL10	IL-10	Gastric gavage	10^9^ bacteria	7 days
		*L. lactis*	pMEC-IL10	*Lactococcus lactis*/pMEC-IL10	IL-10	Gastric gavage	10^9^ bacteria	7 days
Foligné et al. [17]	2006	*L. lactis* MG1363	N.A.	LL-mIL-10	mIL-10	Gastric gavage	10^5^ to 10^9^ CFU	14 days
del Carmen et al. [26]	2014(a)	*L. lactis* MG1363	pValac:il-10	LL-pValac:IL-10	mIL-10	Gastric gavage	10^9^ CFU	14 days
		*L. lactis* MG1363	pGroeESL:il-10	LL-pGroESL:IL-10	mIL-10	Gastric gavage	10^9^ CFU	14 days
Martín et al. [27]	2014	*L. lactis* MG1363	pLB350	LL-IL10	mIL-10	Gastric gavage	10^9^ CFU	10 days
Steidle et al. [16]	2000	*L. lactis*	N.A.	LL-mIL-10	mIL-10	Gastric gavage	2 × 10^7^ CFU or 2 × 10^9^ CFU	2 weeks or 4 weeks
Hamady et al. [23]	2013	*B. ovatus*	N.A.	BO-KGF	KGF-2	Gastric gavage	2 × 10^8^ CFU	5 days
		*B. ovatus*	N.A.	BO-TGF	TGF-β1	Gastric gavage	2 × 10^8^ CFU	5 days
Bermúdez-Humarán et al. [28]	2015	*L. lactis* MG1363	pSEC:mIL-10	LL-IL-10	mIL-10	Gastric gavage	5 × 10^9^ CFU	7 days
		*L. lactis* MG1363	pSEC:mTGF-β	LL-TGF-β	TGF-β	Gastric gavage	5 × 10^9^ CFU	7 days
		*L. lactis* MG1363	pSEC:elafin	*L. lactis* Elafin	Elafin	Gastric gavage	5 × 10^9^ CFU	7 days
		*L. lactis* MG1363	pSEC: mSLPI	*L. lactis* SLPI	SLPI	Gastric gavage	5 × 10^9^ CFU	7 days
Liu et al. [36]	2020	*S. boulardii*	N.A.	N.A.	IL-10	Gastric gavage	10^9^ CFU	5 days
		*S. boulardii*	N.A.	N.A.	TNFR1-ECD	Gastric gavage	10^9^ CFU	5 days
		*S. boulardii*	N.A.	N.A.	AP	Gastric gavage	10^9^ CFU	5 days
		*S. boulardii*	N.A.	N.A.	ANP	Gastric gavage	10^9^ CFU	5 days
		*S. boulardii*	N.A.	N.A.	ANPm	Gastric gavage	10^9^ CFU	5 days
Chiabai et al. [33]	2019	*L. lactis* MG1363 FnBPA + (LL-F)	pValac::anti-TNFα	LL-FT	scFv of anti-TNFα antibody	Gastric gavage	2.0–2.5 × 10^9^ CFU	4 days
Namai et al. [37]	2020	*L. lactis* NZ9000	pNZ8148#2:SEC-IL1Ra	NZ-IL1Ra	mIL-1Ra	Gastric gavage	10^10^ CFU	12 days
Zhang et al. [32]	2018	*E. coli* BL21(DE3)	pET-28a(+)-IL35	*E. coli*/IL-35	IL-35	Gastric gavage	10^10^ CFU	5 days
Wang et al. [35]	2019	*L. lactis* NZ9000	pNZ8148+IL-35	NZ9000/IL-35	mIL-35	Gastric gavage	10^9^ CFU	14 days (3 times weekly)
Zhang et al. [24]	2013	*L. fermentum* I5007	pLK126	*L. fermentum* P126	CAT	Gastric gavage	10^9^ CFU	7 days
Xie et al. [31]	2017	*B. longum* HB15	pBsSOD	*B. longum*-rhMnSOD	MnSOD	Gastric gavage	2 × 10^9^ CFU	7 days
LeBlanc et al. [20]	2011	*L. casei* BL23	pLEM415-mnkat	*Lb. casei* BL23 pLEM415-*mnkat*	CAT	Gastric gavage	10^9^ CFU	24 days
		*L. casei* BL23	pLEM415-sodA	*Lb. casei* BL23 pLEM415-*sodA*	SOD	Gastric gavage	10^9^ CFU	24 days
del Carmen et al. [25]	2014(b)	*S. thermophilus* CRL807	pIL253-sodA	*S. thermophilus* CRL 807:SOD	SOD	Gastric gavage	10^9^ CFU or 3 × 10^10^ CFU	14 days
		*S. thermophilus* CRL807	pIL253-mnkat	*S. thermophilus* CRL 807:CAT	CAT	Gastric gavage	10^9^ CFU or 3 × 10^10^ CFU	14 days
Han et al. [18]	2006	*L. lactis* NZ9800	pNZ8048sodA	*L. lactis* SOD+	SOD	Gastric gavage	10^9^ CFU	8 days
		*L. plantarum* NCIMB8826	pNZ8048sodA	*L. plantarum* SOD+	SOD	Gastric gavage	10^9^ CFU	8 days
Wong et al. [22]	2012	*L. lactis* NZ3900 (N0)	N.A.	N4	mCRAMP	Gastric gavage	10^8^ or 10^10^ CFU	7 days
Li et al. [40]	2021	*L. lactis* NZ9000	pMG36e-Usp45-CRAMP	*L.L-*pMU45CR	CRAMP	Gastric gavage	10^10^ CFU	4 days
		*L. lactis* NZ9000	pNZ8148-Usp45-CRAMP	*L.L-* pNU45CR	CRAMP	Gastric gavage	10^10^ CFU	4 days
Zeng et al. [38]	2020	*L. lactis* NZ9000	pN8148-SHD-5	NZ9000SHD-5	HD-5	Gastric gavage	N.A.	7 days
Esposito et al. [39]	2021(a)	*L. paracasei* F19	pTRKH3-slp-NAPE-PLD	pNAPE-LP	NAPE-PLD	Gastric gavage	0.8–1.2 × 10^8^ CFU	5 days
Park et al. [41]	2021	*E. coli* MG1655	pACYC184-BCD-BUT	MG1655-BCD-BUT	BCD and BUT	Gastric gavage	0.2 × 10^9^ CFU	9 days
		*E. coli* Nissle 1917	pACYC184-BCD-BUT	EcN-BCD-BUT	BCD and BUT	Gastric gavage	0.2 × 10^9^ CFU	9 days
Yan et al. [45]	2021	*E. coli* Nissle 1917	pYX50	EcNL4 (EcNΔ*ldhA*)	3HB	Gastric gavage	5 × 10^10^ cells	7 days
Yoon et al. [19]	2008	*L. casei* BLS	pLUAT-ssMSH	*L. casei*-alpha-MSH	alpha-MSH	Gastric gavage	10^10^ CFU	7 days
Shigemori et al. [30]	2015	*L. lactis* NZ9000	pNZ8148#2:SEC-mHO-1	NZ-HO	rmHO-1	Gastric gavage	5 × 10^9^ CFU	7 days
Praveschotinunt et al. [34]	2019	*E. coli Nissle* 1917	pBbB8k-CsgA-TFF3	PBP8 CsgA-TFF3	Trefoil factors 3	Rectal administration	10^8^ CFU	14 days
Sun et al. [43]	2021	*S. cerevisiae* BY4741	N.A.	*S. cerevisiae 39#*	Lactic acid	Gastric gavage	2 × 10^8^ CFU	7 days
Scott at al [42]	2021	*S. cerevisiae* (CB008)	TM-3 Strain mfa2::HIS3-pFUS1 RROP1	APTM-3	Human P2Y2 purinergic receptor	Gastric gavage	2 × 10^8^ CFU	11 days
		*S. cerevisiae* (CB008)	TM-3 Strain mfa2::HIS3-pFUS1 RROP1	APTM-3	Human P2Y2 purinergic receptor	Gastric gavage	2 × 10^8^ CFU	21 days
		*S. cerevisiae* (CB008)	TM-3 Strain mfa2::HIS3-pFUS1 RROP1	APTM-3	Human P2Y2 purinergic receptor	Gastric gavage	2 × 10^8^ CFU	N.A.
Wang et al. [44]	2021	*E. coli* Nissle 1917	pGEX-4T-1-Sj16-AsBD and pGEX-4T-1-Sj16-GFP-AsBD	EcN-Sj16	Sj16	Gastric gavage	1 × 10^9^ CFU	3 days (Days 0, 4, and 8)
Wei et al. [54]	2016	*B. longum* HB15	pDGMSH	*B. longum*-a-MSH	alpha-MSH	Gastric gavage	2 × 10^10^ CFU	7 days
Wei et al. [55]	2016	*B. longum HB15*	pBDMSH	*B. longum*-a-MSH	alpha-MSH	Gastric gavage	1 × 10^10^ CFU	9 days
Vandenbroucke et al. [46]	2010	*L. lactis* MG1363	N.A.	LL–MT1–MT1	MT1–MT1 Nanobody	Gastric gavage	2 × 10^9^ CFU	14 or 21 days
		*L. lactis* MG1363	N.A.	LL–MT1	MT1 Nanobody	Gastric gavage	3 × 10^9^ CFU	21 days
		*L. lactis* MG1363	pT1mIL10	LL–Mil10	IL-10	Gastric gavage	4 × 10^9^ CFU	14 or 21 days
Liu et al. [53]	2016	*L. lactis* NZ9000	pNZ8148-pIGF-I3	*L. lactis* NZ9000 (pNZ8148-pIGF-I3)	IGF-I	Gastric gavage	4 × 10^12^ CFU	10 days
Zurita-Turk et al. [58]	2020	*L. lactis* MG1363	pValac:il-10	*L. lactis MG1363 FnBPA+ (pValac:il-10)*	IL-10	Gastric gavage	2 × 10^9^ CFU	6 weeks
Qiu et al. [49]	2013	*L. casei* CECT 5276	pIlac-sp-IL10	N.A.	IL-10	Gastric gavage	0.6 × 10^7^ or 0.6 × 10^8^ or 0.6 × 10^9^ CFU	10 days
Yao et al. [48]	2011	*B. longum* NCC 2705	pBBADs-hIL-10	BL-hIL-10	IL-10	Gastric gavage	1.2 × 10^8^ CFU	7 days
Hanson et al. [50]	2014	*L. lactis*	N.A.	LL-IL-27	IL-27	Gastric gavage	2 × 10^8^ CFU	14 days
Whelan et al. [52]	2014	*E. coli Nissle* 1917	pMU13 -AvCys	EcN-AvCys	Nematode cystatin	Gastric gavage	2 × 10^9^ CFU	4 days
Breyner et al. [57]	2019	*L. lactis* NZ9000	pSEC:PAP	LL-PAP	PAP	Gastric gavage	5 × 10^9^ CFU	9 or 17 days
Liu et al. [56]	2018	*B. longum* HB25	pBDMnSOD	*B. longum*-PEP-1-rhMn-SOD	rhMn-SOD	Gastric gavage	2 × 10^9^ CFU	7 days
Hou et al. [51]	2014	*L. fermentum* I5007	pMF009	*L. fermentum* (pMF009)	SOD	Gastric gavage	5 × 10^9^ CFU	6 days
Watterlot et al. [47]	2010	*L. casei*	pILKSsodA	*Lb. casei* pILKSsodA	SOD	Gastric gavage	5 × 10^9^ CFU	9 days
		*L. casei*	pVE3874	*Lb. casei* BL23 pVE3874	CAT	Gastric gavage	5 × 10^9^ CFU	9 days
Aubry et al. [60]	2015	*L. lactis* MG1363	pGroESL-TSLP	LL-TSLP	TSLP	Gastric gavage	1–5 × 10^9^ CFU	4 or 12 or 17 days
Foligne et al. [59]	2007	*L. lactis* MG1363	pMEC237	LL-LcrV	Immunomodulatory Yersinia LcrV Protein	Gastric gavage	2 × 10^8^ CFU	5 days

## Data Availability

The data underlying this article are available in the article and in its online Supplementary Material.

## Supplementary Materials

The following supporting information can be downloaded at: https://www.mdpi.com/article/10.3390/nu15071566/s1.

## Abbreviations

CFU	Colony-forming unit
DAI	Disease activity index
GIT	Gastrointestinal tract
CD	Crohn’s disease
UC	Ulcerative colitis
DSS	Dextran sulphate sodium
TNBS	Trinitrobenzene sulfonic acid
DNBS	Dinitro-benzenesulfonic-acid
Gm probiotics	Genetically modified probiotics
IBD	Inflammatory bowel disease
MPO	Myeloperoxidase
SCFAs	Short-chain fatty acids
